# Assessing Digital Determinants of Health and Their Impact as Part of Strategic Health Planning in Catalonia: Modified Delphi Study

**DOI:** 10.2196/93840

**Published:** 2026-10-29

**Authors:** Mireia Cano, Aïna Fuster-Casanovas, Marta Aymerich, Meritxell Davins, Carme Carrion

**Affiliations:** 1 eHealth Lab Research Group Universitat Oberta de Catalunya Barcelona, Catalonia Spain; 2 Unitat d'Estudi del Medicament Institut Universitari d'Investigació en Atenció Primària (IDIAPJGol) Barcelona, Catalonia Spain; 3 Innovation, Health Economics and Digital Transformation Research Group Institut d'Investigació en Ciències de la Salut Germans Trias i Pujol Badalona, Catalonia Spain; 4 eHealth Lab Research Group, School of Medicine Universitat de Girona Girona, Catalonia Spain; 5 Network for Research on Chronicity, Primary Care, and Health Promotion (RICAPPS) Barcelona Spain

**Keywords:** digital determinants of health, digital health, health equity, health planning, Catalonia, Catalan health system

## Abstract

**Background:**

Digital health technologies are transforming health care by improving access, efficiency, and quality of care. However, recent studies highlight that digitalization can unintentionally reinforce or deepen existing social and health inequalities, as not all population groups benefit equally from digital progress. The concept of digital determinants of health (DDoH) has emerged to better capture how digital factors shape health opportunities and outcomes. The systematic incorporation of DDoH and other health determinants shaped by digitalization into strategic planning remains limited. As a result, health systems may lack structured approaches to identify them and anticipate their equity implications.

**Objective:**

This study aimed to identify which health determinants are considered most important in the Catalan context and to qualitatively explore their inclusion in existing regional strategies and policy frameworks in Catalonia.

**Methods:**

We conducted a modified Delphi study with experts from the Catalan health and digital ecosystem. The list of determinants was derived from a scoping review, which identified 127 determinants that emerged or changed with digitalization. Experts living and working in Catalonia, with experience in digital health, public health, health care delivery, social or DDoH, or health equity, were recruited using snowball sampling. Participants rated each determinant for importance using a 5-point Likert scale across 2 rounds, followed by a final online consensus meeting. For each determinant, the median (IQR) and the proportion of experts rating it as 4 or 5 were calculated, and items were classified into high, moderate, or low relevance according to predefined consensus criteria.

**Results:**

A total of 43 experts participated in the first Delphi round, and 31 completed the second round. Of the 127 assessed determinants, 20 (15.7%) reached high relevance for the Catalan context, including 15 digital, 4 social, and 1 political determinant. Overall, the most important determinants focused on enabling the effective and equitable use of digital health, particularly access, skills, system-level digital capacity and governance, inclusive design, and the responsible use of AI. Comparison with the original global Delphi study showed substantial overlap, but also revealed context-specific priorities.

**Conclusions:**

This study demonstrates the value of systematically contextualizing DDoH and other health determinants shaped by digitalization to inform strategic health planning in digitally mature public health systems. The findings show that international digital health agendas require regional reprioritization to reflect local infrastructure, governance arrangements and equity challenges. The results suggest that, beyond technological capacity, governance and equity considerations should become central to digital health planning. The proposed approach offers a replicable framework to support more anticipatory, coordinated and equity-oriented digital health strategies.

## Introduction

In recent years, the rapid expansion of digital technologies has positioned digital health as a central driver of the transformation of health care systems worldwide. Digital tools have created new opportunities to improve efficiency, access, quality of care, and personalization [1-7]. The strategic relevance of digital transformation is also reflected in the fact that, according to the World Health Organization’s (WHO’s) 2025 update [8], up to 129 countries have developed specific national digital health strategies to guide the adoption, governance, and scaling of these technologies. Across Europe, these strategies prioritize interoperability, data governance, user-centered design, cybersecurity, and the expansion of digital services [9-13].

However, the success of these national digital health strategies does not depend solely on technological development or system-level digital capacity. It is widely known that health outcomes are still strongly influenced by the social conditions captured in the social determinants of health (SDoH) framework, which can either protect against or intensify health risks. In this context, growing evidence indicates that the benefits of digitalization are not equitably distributed, and digital transformation can unintentionally reproduce or exacerbate those existing social and health inequalities [14,15]. Although most national digital health strategies acknowledge challenges such as digital divides, uneven skills, or barriers to adoption, these concerns are rarely translated into structured mechanisms to assess how digital factors may reinforce or mitigate inequities [9-12].

This growing awareness has led to increasing recognition of digital factors as determinants of health in their own right [14], with a strong capacity to reshape, amplify, or modify existing determinants and to influence how social, economic, political, and commercial factors operate and interact [16]. In this context, the concept of digital determinants of health (DDoH) has emerged to capture how digital factors shape health opportunities and outcomes, and is increasingly used in reports from international organizations and high-impact journals [14,16-20]. This wider adoption suggests that DDoH justify being considered as a distinct, though closely related, category rather than being subsumed under traditional SDoH labels, while still remaining anchored in the broader SDoH framework. However, important gaps remain in the literature [21]. There is still no consensus on how DDoH should be defined, where to draw the boundaries between digital and traditional SDoH, or which specific variables ought to be considered DDoH in different contexts.

In this regard, Duric et al [21] argue that the definition proposed by van Kessel et al [16] currently offers the most comprehensive and operational description of DDoH, defining them as “any factor rooted in, contingent on, or inextricably linked to the digital world that can directly or indirectly influence health or well-being. These determinants can change how health care is delivered to improve health, modify existing relationships between social, political, or commercial determinants and health, or create entirely new ways to influence individual or population health” [16]. van Kessel et al [16] identified 127 health determinants that have emerged or evolved with digitalization (37 digital, 33 social, 33 commercial and economic, and 24 political) [16]. Their scoping review reflects the extensive influence of digitalization on health, but also poses challenges for policymakers and planners, who must decide which determinants deserve priority attention in specific health system contexts. Their Delphi exercise highlighted 30 determinants requiring urgent policy attention, including internet access, digital literacy, interoperability, and inclusive design, among others. However, the authors also noted that translating these priorities into local policy contexts is difficult given regional variations in digital infrastructure, social norms, and health system organization. Contextualizing these determinants is essential to ensure that digital transformation efforts are aligned with local needs, capacities, and equity considerations.

Catalonia represents a particularly relevant case for examining the role of DDoH alongside other health determinants shaped by digitalization, as it combines a universal public health system with a high level of digital health maturity. In fact, the region has long been considered a leader in digital health innovation within Europe, with an established ecosystem of digital services, a strong IT Infrastructure, large-scale interoperability initiatives, and successive strategic plans promoting data-driven and person-centered care [22-26]. Key initiatives include the electronic health records (EHRs), digital medical imaging, the Rec@t electronic prescription system, multiple telemedicine programs, and La Meva Salut (My Health, translated into English), the official patient portal of the Catalan public health care system [23,27-30]. Recently, this commitment was further consolidated in the Catalan Health Plan 2021-2025, which established digital transformation as a core strategic axis [24].

Despite Catalonia’s advanced digital maturity, persistent social and territorial inequalities in digital access, digital use, skills, and health outcomes have been documented, suggesting that not all population groups benefit equally from technological progress [31-35]. Catalan strategic documents acknowledge related concerns, such as the digital divide, uneven levels of digital literacy, and the possibility that digital solutions may unintentionally reinforce existing inequalities [24]. However, while these strategies recognize elements aligned with the umbrella of DDoH, they do not yet adopt a structured, comprehensive approach that systematically considers the full spectrum of digital factors that may influence health. As a result, some digital factors with potential implications for equity may not be fully captured or consistently prioritized. This combination of strong digital capacity but uneven digital benefits makes Catalonia an informative setting for contextualizing DDoH and identifying both the most relevant DDoH and other health determinants shaped or modified by their influence, thereby supporting evidence-informed policy decisions and strengthening digital governance to narrow, rather than widen, existing health inequities.

Therefore, the objective of this study was to determine which of the 127 health determinants that have emerged or evolved with digitalization were considered more important in Catalonia, by replicating and adapting the Delphi approach used by van Kessel et al [16] and to qualitatively explore how these determinants are reflected in existing regional strategies and policy frameworks. In doing so, the study (1) provides a regional prioritization of health determinants that have emerged or evolved with digitalization, (2) compares these regional priorities with the global Delphi results, (3) examines how the most prioritized determinants are addressed in Catalan policy and other relevant documents, and (4) explores how these priorities can inform strategic health planning.

## Methods

### Study Design

This study followed a modified Delphi design [36], a structured multiround consensus method commonly used to address complex topics when empirical evidence is limited.

### Ethical Considerations

The study was conducted in accordance with the Declaration of Helsinki [37] and approved by the Ethics Committee of the Open University of Catalonia (CE25-TE04). All participants provided informed consent. Data were collected anonymously and no financial compensation was provided for participation.

### Study Population

Eligible participants were professionals with expertise or practical experience in digital health, public health, health care delivery, SDoH, DDoH, or health equity, including clinicians, managers, policy makers, technical experts, entrepreneurs, and researchers. Participants were required to have resided in Catalonia for at least 12 months, be fluent in Catalan, and be willing to actively participate. Efforts were made to ensure diversity in gender, geography, and professional background. Individuals not meeting these criteria were excluded.

### Sample Size and Participants Recruitment

Given the qualitative nature of the modified Delphi process, no formal sample size calculation was performed. Although the literature suggests that panels of around 10 experts can be sufficient for Delphi studies [38], we aimed to recruit at least 35 participants to align with the original study [16] and to ensure diversity across stakeholder groups.

Participants were recruited using a snowball sampling strategy, a nonprobabilistic approach commonly used in qualitative research to reach expert populations [39]. The research team initially contacted 8 experts as seed participants, drawn from key institutions within the Catalan health system and identified through existing professional networks and prior collaborations. These initial participants were purposely selected based on their expertise in digital health, public health, health care delivery, or health equity. The seed participants were then invited to recommend additional experts within their professional networks, in line with a snowball sampling strategy. During recruitment, the research team monitored gender, professional profile, and health region using a recruitment matrix to promote a sufficiently diverse and reasonably balanced sample across these characteristics. This process resulted in the identification of 80 potential participants. All identified candidates were subsequently contacted by email and received a brief explanation of the study objectives and participation requirements. To minimize potential bias in the Delphi assessment, the list of determinants was not shared in advance. Data collection took place between September and December 2025.

### Study Variables

#### Demographic Variables

Participants were asked to complete a part of the survey that collected various demographic data: age, gender, educational level, professional background, actual job position, sector, years of experience in the professional field in general, years of experience in public health, years of experience in digital health, self-rated expertise on SDoH, self-rated expertise on DDoH, and work health region (in Catalonia, the public health system is organized into territorial health regions defined by the regional health administration, which do not necessarily coincide with other administrative or political boundaries). More details are shown in Multimedia Appendix 1.

#### Main Study Variable

The primary outcome of this study was the level of importance assigned to each determinant by the expert panel. Experts rated each determinant using a 5-point Likert scale (1=not urgent, 2=somewhat urgent, 3=fairly urgent, 4=urgent, and 5=very urgent), responding to the question: “Recognizing that all health determinants are important to address, how important is it for this determinant to be taken into account in the Catalonia?”

### Data Collection and Study Procedure

The study was structured in 4 phases. The overall process is illustrated in Figure 1.

1. Phase 1: Defining the determinants set. The initial list of determinants was derived from a recent systematic review and expert consensus process conducted by van Kessel et al [16], which identified 127 determinants of health across 4 domains (digital, social, commercial and economic, and political). These determinants, organized into the thematic clusters proposed by the original authors, provided a validated starting point for the Delphi process.
2. Phase 2: Delphi consultation rounds. In the first round, participants received an online questionnaire via the Qualtrics platform (Qualtrics XM Platform), including informed consent, demographic questions, and the list of determinants to be rated on a 5-point Likert scale. Participants were given up to 3 days to complete each round so that they could accommodate the time required to rate a large number of items alongside their routine professional responsibilities. After analysis of round 1, only determinants classified as moderate relevance were included in round 2 for reevaluation, following the same approach used by van Kessel et al [16]. This strategy was chosen both to ensure methodological comparability with the original global Delphi and to limit respondent burden in the second round by avoiding the rerating of items for which there was already consensus on high or low relevance. Only fully completed questionnaires were included in the analysis for each round, in order to maximize the completeness and basic quality of the data for all rated determinants.
3. Phase 3: Consensus meeting and final analysis. Following analysis of round 2, all experts who had expressed interest in participating in the final round were invited to attend a final consensus meeting to validate and clarify the round 1 and round 2 results, and to confirm whether any proposal to modify the list or classification of determinants emerged.
4. Phase 4: Narrative review of regional policy and strategy documents. We conducted a narrative documentary review of key regional documents related to digital health and health equity in Catalonia. Relevant documents and reports were first identified through the official websites of key institutions involved in health planning and digital transformation, including the Catalan Department of Health, the Healthcare Quality and Assessment Agency of Catalonia (AQuAS), the TIC Salut i Social Foundation, the Department of Digital Policies and the Catalan Health Institute; and where appropriate, documents from Spanish national bodies and international organizations (eg, the Ministry of Health, data protection authorities, the European Commission, the WHO). When more specific technical or contextual information was needed, this search was complemented by targeted searches in scientific databases and Google using free‑text terms in Catalan, Spanish and English. The review focused on publicly available policy, strategy and planning documents that were considered directly relevant to the study aims, such as health plans, digital health strategies, equity policies and major reports. Rather than following a formal systematic or thematic review approach, the documents were read iteratively and used to extract information most relevant for interpreting the Delphi findings and situating them within the Catalan policy context.

**Figure 1:**
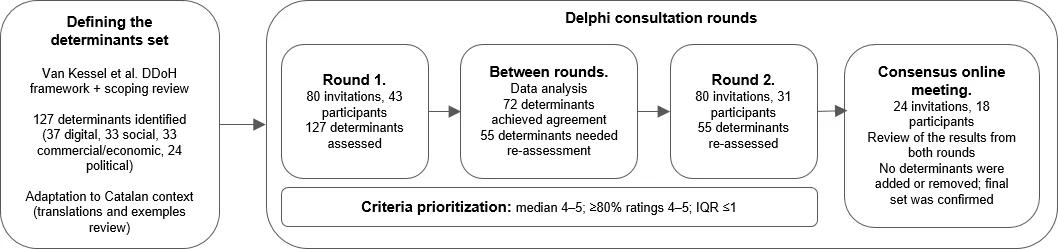
Overview of the study design and methodological phases. DDoH: digital determinants of health.

### Data Analysis

Data were collected using Qualtrics-XM (Qualtrics) and analyzed with Jamovi v.2.6 for Windows (Jamovi). As no personally identifiable information was collected, participants remained anonymous, and no coding system was required. Demographic characteristics were summarized using descriptive statistics and presented in tables.

Each determinant was analyzed using the same consensus criteria applied by van Kessel et al [16], which were used to prioritize health determinants at a global level. For each item, the median score and IQR were calculated to assess central tendency and agreement, and the importance percentage was calculated, which was defined as the proportion of participants who rated an item as 4 or 5 on the 5-point Likert scale. Based on these indicators, determinants were classified as: high relevance (median 4-5, importance ≥80%, IQR ≤1), moderate relevance (median ≤4 and importance 50%-79%), or low relevance (median <4, importance <50%, and IQR>1). These thresholds were defined a priori to maximize comparability with the original study and are also in line with commonly used Delphi practice, which typically combines measures of central tendency, dispersion, and approximately 75%-80% agreement on the highest response categories to define consensus [40].

### Data Confidentiality

The data used in this study were anonymous and were stored on the servers of the Open University of Catalonia, which acted as the data controller. Throughout the process, all necessary procedures were implemented to ensure the protection of the confidentiality of the information.

## Results

### Participants

Table 1 summarizes the sociodemographic and professional characteristics of the participating experts. Of the 80 experts invited to participate, 43 completed round 1, corresponding to an acceptance rate of 53.8%. Of these, 31 participated in round 2, corresponding to a dropout rate of 27.9% between rounds. Overall, the panel remained relatively stable across rounds and reflected a high level of professional qualification and disciplinary diversity.

**Table 1:** Sociodemographic and professional characteristics of experts participating in the 3 rounds of the study.

Sociodemographic characteristics			Experts in first round (n=43)		Experts in second round (n=31)
Age (years), mean (SD)			44.6 (10)		46.8 (10.5)
**Sex, n (%)**					
	Female	25 (58.1)		17 (54.8)	
	Male	17 (39.5)		14 (45.2)	
	Others	1 (2.3)		—^a^	
**Educational level, n (%)**					
	Bachelor’s degree	3 (7)		2 (6.5)	
	Master’s degree	17 (39.5)		11 (35.5)	
	PhD degree	23 (53.5)		18 (58.1)	
**Professional background or title, n (%)**					
	Medicine	16 (37.2)		13 (41.9)	
	Nursing	5 (11.6)		2 (6.5)	
	Engineering	4 (9.3)		2 (6.5)	
	Psychology	4 (9.3)		1 (3.2)	
	Physiotherapy	2 (4.7)		1 (3.2)	
	Pharmacy	2 (4.7)		2 (6.5)	
	Economics	2 (4.7)		—	
	Business administration	1 (2.3)		2 (6.5)	
	Other	7 (16.1)		8 (25.8)	
**Current professional role, n (%)**					
	Leadership or Management	20 (45.5)		14 (45.2)	
	Research or academia	14 (31.8)		9 (29)	
	Clinical role	10 (22.7)		8 (25.8)	
**Sector, n (%)**					
	Public health care sector	26 (58.1)		19 (61.3)	
	Private health care sector	2 (4.7)		1 (3.2)	
	Mixed public-private health care sector	3 (7)		1 (3.2)	
	Research - University	12 (27.9)		10 (32.3)	
**Health region, n (%)**					
	Alt Pirineu i Aran	1 (2.3)		—	
	Lleida	2 (4.7)		3 (9.7)	
	Camp de Tarragona	—		—	
	Terres de l'Ebre	—		—	
	Central Catalonia	3 (7)		3 (9.7)	
	Girona	5 (11.6)		4 (12.9)	
	Barcelona City	6 (14)		4 (12.9)	
	Barcelona North Metropolitan	10 (23.3)		4 (12.9)	
	Barcelona South Metropolitan	1 (2.3)		1 (3.2)	
	Penedès	—		—	
	Catalonia^b^	15 (34.9)		12 (38.7)	
**Years of experience, mean (SD)**					
	Years of experience in health care	17.4 (10.8)		18.3 (11.2)	
	Years of experience in public health	13.6 (12.4)		13.5 (11.4)	
	Years of experience in digital health	5.53 (6.5)		7.29 (7)	
**Self-rated expertise, mean (SD)**					
	Self-rated expertise on SDoH^c^	7.63 (1.3)		7.81 (1.3)	
	Self-rated expertise on DDoH^d^	6.44 (2)		7.26 (1.7)	

^a^Not applicable.

^b^Participants whose professional role or responsibilities span most or all health regions of Catalonia.

^c^SDoH: social determinants of health.

^d^DDoH: digital determinants of health.

Participants had a mean age of 44.6 (SD 10) years in round 1 and 46.8 (SD 10.5) years in round 2. In both rounds, most participants identified as female (25/43, 58.1% in round 1 and 17/31, 54.8% in round 2). Educational level was high: in round 1, 39.5% (17/43) held a master’s degree and 53.5% (23/43) a PhD or higher, while in round 2, 35.5% (11/31) held a master’s degree and 58.1% (18/31) a PhD. Medicine was the most frequently represented academic background, followed by nursing, engineering, and psychology, while less-represented disciplines were grouped as “other” in Table 1.

Nearly half of the panel held leadership or management roles (20/43, 45.5% in round 1; 14/31, 45.2% in round 2), followed by research or academic and clinical roles. Most participants were affiliated with the public health sector (26/43, 58.1% and 19/31, 61.3%, respectively). Experts were drawn from several health regions in Catalonia, although Camp de Tarragona, Penedès, and Terres de l’Ebre were not represented.

Participants also reported substantial experience in health care, public health, and digital health, as well as high self-rated expertise in both SDoH and DDoH. In the final online consensus meeting, 18 of the 24 experts who had previously indicated their willingness to participate in this final phase attended, and no additional determinants were added or removed.

### Key Health Determinants

In the first Delphi round, all 127 variables were assessed, with 72 reaching consensus (59 classified as low relevance and 13 as high relevance). The remaining 55 variables, classified as moderate relevance in the first round, were re-evaluated in the second round. Following this reassessment, 7 were reclassified as high, 42 as moderate and 6 as low, resulting in a final distribution of 20 high‑, 42 moderate‑ and 65 low‑relevance determinants across domains. Round 1 and round 2 results were presented in a final online validation meeting attended by 18 of 24 invited experts; after reviewing the descriptive results, participants agreed to maintain the existing classification of determinants, and no changes were proposed. Figure 2 shows the number of determinants classified as high, moderate, and low relevance by domain across Delphi rounds and in the final result.

Of the 20 high‑relevance determinants (15.7% of the total), 15 belonged to the digital domain, 4 to the social domain, and 1 to the political domain, while no commercial or economic determinants met the criteria for high relevance. The final list of highly relevant determinants is presented in Table 2, while the complete set of variables and consensus outcomes is available in Multimedia Appendix 2.

When compared with the determinants globally prioritized by van Kessel et al [16], the degree of overlap varied across domains. In the digital domain, 14 of the 20 global priorities also reached high relevance in Catalonia, 6 did not, and 1 additional determinant (personal customizability) emerged. In the social domain, 2 of the 6 overlapped (inclusive design and good practice design), 4 did not, and 2 additional determinants emerged (health literacy and urbanicity). In the political domain, only 1 of the 4 overlapped, the remaining 3 did not, and none were newly added. No commercial or economic determinant reached high relevance in either study.

Overall, 17 of the 20 determinants prioritized in Catalonia overlapped with the 30 identified in the global Delphi, while 3 were unique to the Catalan exercise. Conversely, 13 globally highlighted determinants did not reach high priority in our process, including moderation of harmful content and misinformation; explainability; mis- and/or disinformation policy; outcomes, utility, and value sharing; data culture; regulatory mandate; health and social care policy; socioeconomic inequalities; access to health and social services; health and disability status; ownership of technology; digitalization agenda; and open and transparent decision-making.

**Figure 2:**
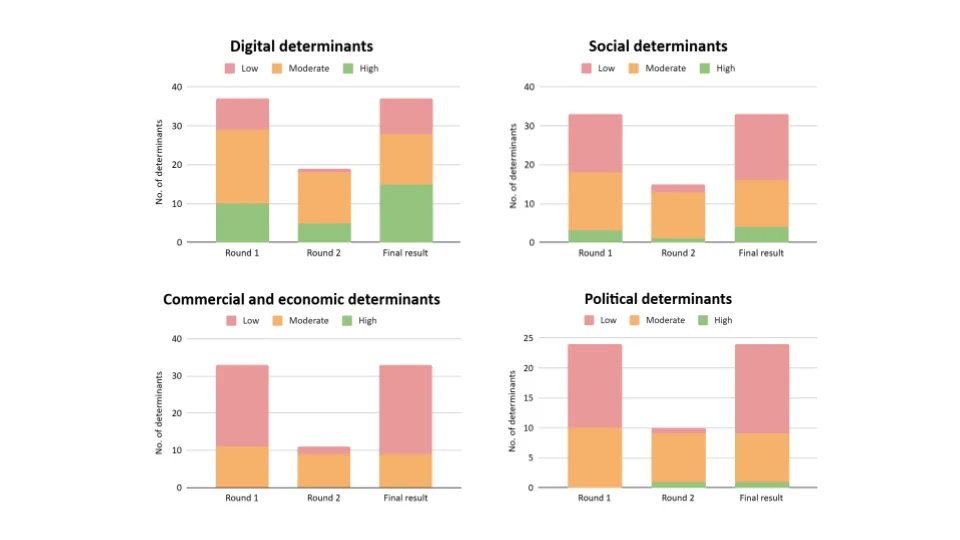
Number of determinants classified as high, moderate and low relevance by domain across Delphi rounds and in the final result.

**Table 2:** The 20 determinants that reached consensus and were rated as highly important in the Catalan context.

Digital ecosystem domain, societal category, and health determinant				Importance				
			Median (IQR)		Number of respondents/total respondents (%)		Consensus round	
**Digital domain**								
	**Person-specific determinants**							
		Device and software availability	4 (1)		37/43 (86)		Round 1	
		Digital literacy	4 (1)		35/43 (81)		Round 1	
		Internet access and connectivity	5 (1)		26/31 (84)		Round 2	
	**Community determinants**							
		Data and digital capacity	4 (1)		36/43 (84)		Round 1	
	**Technology-related determinants**							
		Model accuracy and algorithmic validation	4 (1)		35/43 (81)		Round 1	
		Personal customizability	4 (1)		36/43 (84)		Round 1	
		Data and digital interoperability	4 (1)		36/43 (84)		Round 1	
		Security settings and features	4 (1)		36/43 (88)		Round 1	
	**Policy determinants**							
		Privacy and security policy	5 (1)		35/43 (81)		Round 1	
		AI validation, transparency, explainability, accountability, and ethics	5 (1)		27/31 (87)		Round 2	
		Data consent policy	5 (1)		27/31 (87)		Round 2	
		Access and sharing policy	4 (1)		26/31 (84)		Round 2	
	**Political, economic, societal and cultural determinants**							
		Digital divides	4 (1)		36/43 (84)		Round 1	
		Digital public infrastructure	4 (1)		36/43 (84)		Round 1	
		Data governance and ethics	5 (1)		26/31 (84)		Round 2	
**Social domain**								
	**Person-specific determinants**							
		Health literacy	4 (1)		40/43 (93)		Round 1	
		Urbanicity	4 (0)		30/31 (97)		Round 2	
	**Technology-related determinants**							
		Inclusive design	4 (1)		36/43 (84)		Round 1	
		Good practice design	4 (1)		38/43 (88)		Round 1	
**Political domain**								
	**Political, economic, societal and cultural determinants**							
		Accountability and transparency of commercial interests	4 (1)		25/31 (81)		Round 2	

## Discussion

### Principal Findings

In recent years, digitalization has transformed almost every domain of society, including how health systems are organized and how care is delivered [8]. This transformation has introduced new digitally mediated factors into the health arena and has also modified the way traditional determinants of health operate [14,16,19,20]. In response, academic and policy actors have increasingly used the term DDoH to describe how digital factors influence health and health equity [14,16]. This emerging terminology positions DDoH as a distinct but interdependent domain within the broader determinants of health, consolidating previously fragmented digital variables into a more coherent framework and signaling their growing relevance as a global policy priority. Nevertheless, this framing is not yet settled, as no consensus exists on how DDoH should be defined, nor on whether they constitute a distinct domain of health determinants or a subset of the SDOH [21].

Beyond these conceptual debates, a more practical gap persists: although DDoH are increasingly recognized internationally as key drivers of health opportunities and equity [24,31,34], their effects are rarely assessed systematically at the regional or health-system level [14,16,41]. As a result, health systems often lack structured mechanisms to identify which DDoH and other health determinants shaped by digitalization are most relevant to their own context and to translate that knowledge into planning and policy. To our knowledge, this is the first study to replicate and adapt the global Delphi exercise to a specific health system context, systematically identifying which of the 127 determinants are most relevant for health planning in Catalonia [16].

In doing so, our findings reveal both alignment and divergence with the global prioritization [16], reflecting a combination of global challenges and context-specific priorities shaped by Catalonia’s territorial, social and organizational characteristics. When examined more closely, the strongest convergence is located within the digital domain, whereas the greatest divergences are concentrated in the social and political domains. This domain‑level pattern reinforces the idea that DDoH may combine a broadly universal core, capturing those conditions that are necessary for digital health to function at all, regardless of setting, such as connectivity, device availability, basic digital skills, interoperable data infrastructures and robust privacy and security frameworks; alongside more context-sensitive layers.

Although this interpretation is derived from our study, it will be important to examine whether similar patterns emerge in other countries and whether DDoH are consistently prioritized above other domains; if so, this would support the view that some DDoH could operate as instrumental conditions that could determine whether individuals and groups can effectively benefit from digital health interventions or, conversely, remain structurally excluded from them. In legal and human rights debates, comparable elements, for example the right to internet access, have been described as instrumental human rights [42] because they enable the exercise of other fundamental rights such as education, work or political participation. A similar logic would apply in health when access to services would be channeled primarily through online pathways (eg, when booking appointments is only possible via digital platforms), effectively turning some DDoH into gatekeeping conditions for entering the health system at all.

On the other hand, the context‑specific layers appear, in our study, more prominently in the social and political domains, where the importance of those determinants would depend on how digitalization intersects with existing health and social protection systems, regulatory traditions and policy priorities. In Catalonia, this may be partly explained by the characteristics of a universal health system, a strong public service orientation and relatively mature regulatory and governance frameworks [24,43,44]. Notably, both in our Delphi and in the original global exercise [16], commercial and economic determinants, and political determinants were underrepresented among high-relevance items. This pattern may suggest that experts tended to prioritize domains perceived as more directly actionable within current health planning processes. However, it may also reflect the composition of our panel, which consisted mainly of experienced health system professionals and managers with long trajectories in health and public health, while commercial and broader political sectors were comparatively less represented.

Finally, the results of this study highlight the strongly interconnected nature of health determinants, indicating that all of them operate as part of a complex and interdependent system rather than as isolated elements. As a result, health inequalities may not simply accumulate, but grow nonlinearly when multiple determinants intersect, as already shown in the literature [14,17,20,41,45].

For this reason, the discussion is organized around analytical axes rather than individual determinants. These axes were developed by the research team through a consensus-based interpretive process after discussion of the conceptual relationships among determinants. This strategy was used to avoid repetitive, fragmented interpretation of determinants that addressed closely related issues. The resulting axes should therefore be understood as analytical groupings created to support interpretation and discussion, and in no case are they intended as a new formal classification of determinants. Figure 3 illustrates how the variables were distributed across the analytical axes.

**Figure 3:**
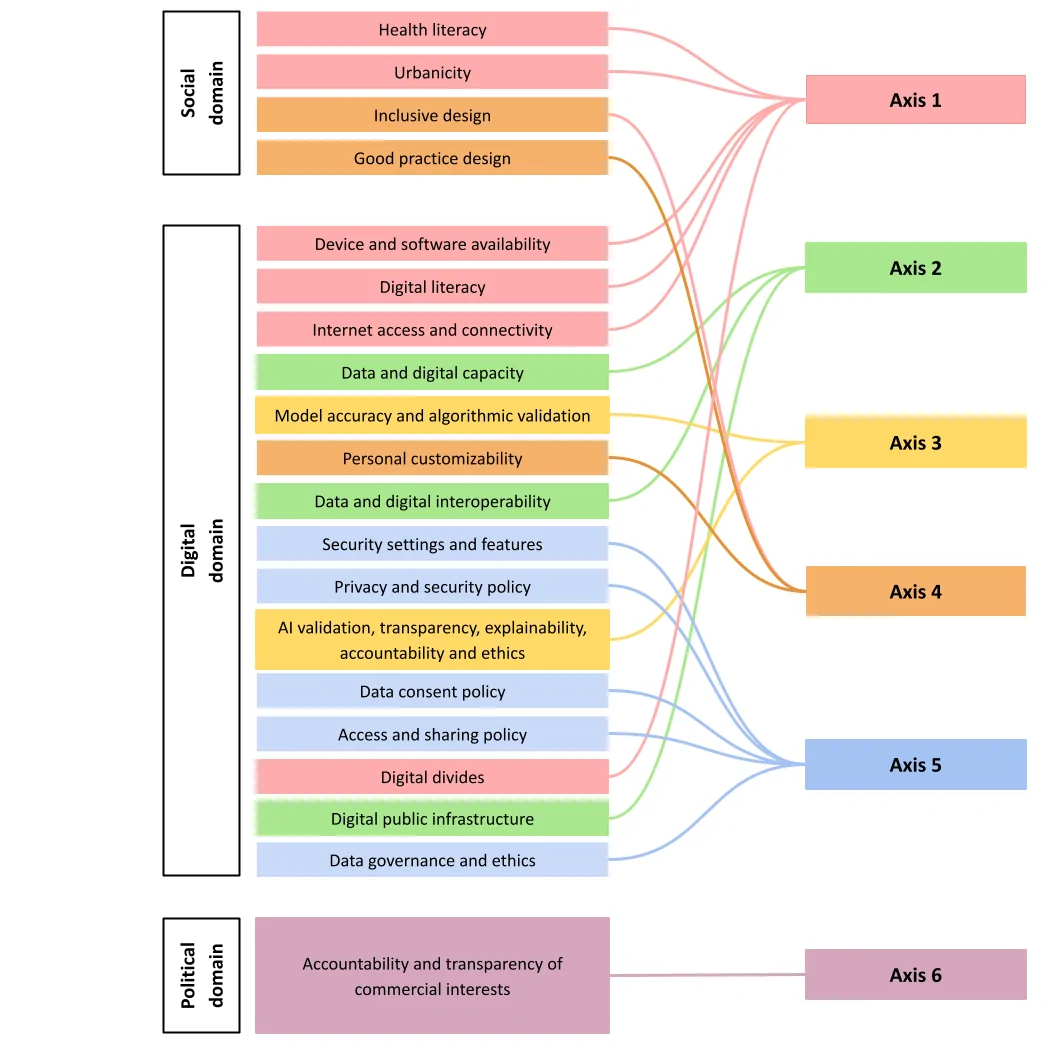
Distribution of variables across conceptually defined analytical axes.

Building on this structure, the axis‑based discussion was informed by a narrative review of regional and related policy documents, which ultimately included 57 references out of approximately 95 documents screened, most of them published in the last 6 years. This body of evidence is drawn primarily from Catalan public institutions, complemented by Spanish and international organizations and recent literature on digital health and equity, and was used to contextualize each axis without aiming to provide an exhaustive policy review.

### Axis 1

This axis includes device and software availability, digital literacy, internet access and connectivity, digital divides, urbanicity and health literacy. As digital interventions become embedded across care pathways, effective use depends on the alignment between individual capabilities, contextual conditions and service design.

Connectivity illustrates these dynamics clearly. Despite high average 5G coverage in Catalonia (94.6%), marked territorial disparities persist between urban and rural or mountainous areas [31], and are acknowledged in official regional strategic plans as a priority for digital inclusion and equitable access to public digital services [46]. In practice, these disparities have important implications for how digital health services are experienced: in rural settings, where connectivity gaps often coincide with longer distances to health facilities and fewer in-person support options, digital tools may substitute rather than complement face-to-face care, turning these infrastructure limitations into structural barriers and increasing the risk of exclusion [26,47-49].

A similar logic applies to device and software availability. Despite almost universal mobile phone access in Catalonia (99.4%), reports show that inequalities emerge primarily at the level of use rather than ownership. [31,50]. These disparities could be closely linked to digital health literacy levels. Although digital health literacy is not directly measured, indirect indicators can highlight substantial disparities. Lower uptake of La Meva Salut among older adults, particularly in rural areas and among those aged ≥75 years, mirrors broader sociodemographic and territorial gradients in internet use and digital competence [31,46,50], consistent with evidence from Spain [51].

At the policy level, the Health Plan for Catalonia and the Digital Health Strategy recognize the strengthening of both digital and health literacy among citizens and professionals as strategic priorities [24,26]. However, their translation into practice remains uneven, although regional institutional frameworks explicitly call for reducing structural barriers and preserving in-person support mechanisms to strengthen digital and health literacy among vulnerable groups [52], many initiatives still rely predominantly on online delivery formats that may unintentionally limit their participation [53-56].

Overall, this axis reinforces that “digital divides” reflect the cumulative interaction of infrastructure, skills, and territorial constraints rather than isolated barriers. As digital services expand [52,57-59], these determinants may cluster and amplify inequities in access to health care, quality of care, and health outcomes [45,60], underscoring the need for targeted actions.

### Axis 2

This axis includes data and digital capacity, data and digital interoperability, and digital public infrastructure and examines the capacity of health systems to use the technology in effective, equitable, and sustainable ways.

Catalonia has developed one of the most consolidated health data ecosystems in Southern Europe through sustained public investment in health information technologies and digital integration across the Integrated Public Healthcare System of Catalonia (SISCAT) [23,61]. This includes the widespread implementation of EHRs across publicly funded care settings and an interoperable infrastructure designed to support information continuity and reduce fragmentation [23,62]. Its development has been supported by the systematic adoption of international clinical and technical standards, enabling semantic consistency and scalable data exchange [63]. In parallel, Catalonia has developed substantial research-oriented data platforms illustrating high system-level data exploitation capacity. Notably, the Information System for the Development of Research in Primary Care (SIDIAP) integrates large-scale, pseudonymized primary care data and is widely used for population-based and epidemiological research [64].

However, despite this high level of digital maturity, policy and empirical evidence indicate that digitalization has not fully translated into integrated, longitudinal and person-centered care. The central challenge is therefore less about system availability than about governance, integration and embedding in routine practice [26]. Empirical studies consistently show that the use of shared interoperable infrastructures such as EHRs remains uneven, constrained by usability issues, partial workflow integration, data quality heterogeneity and variable digital competence among professionals [27,65,66]. In response, the current SISCAT strategy shifts the focus from digitalization as simple documentation toward digital systems as active enablers of continuity and coordination, framed within a citizen-centered information systems model. A central element of this reorientation is the new EHR, Historial Electrònic de Salut (HES), conceived as a single, interoperable health and social care record based on an open platform model, with full implementation across selected providers planned for 2031. This direction aligns with the adoption of openEHR-based architectures, promoted in Catalonia to address fragmentation, support longitudinal records and reduce vendor lock-in [67].

However, these changes do not automatically translate into effective or equitable use in practice. For example, despite sustained investment in AI and data-driven initiatives, much of this capacity remains concentrated in innovation settings, with limited diffusion into routine care [68-70]. Although current strategies emphasize strengthening digital competencies among professionals as a prerequisite for embedding these systems into routine practice, alongside analytics and decision-support tools for more proactive and coordinated care, these competencies remain unevenly distributed and insufficiently embedded within public health organizations, constraining equitable uptake across care settings and territories [26].

Finally, system-level digital capacity is further shaped by broader digital infrastructures linked to Catalonia’s wider digital transformation, such as large-scale data centers (mainly private initiatives) and high-performance computing facilities supporting data-intensive research and AI applications [71,72]. Although not health-specific, these infrastructures condition how health data are processed at scale and may create dependencies within public–private configurations, raising governance questions regarding alignment with priorities of the public health care system and equitable access to resources [73].

### Axis 3

This axis includes the variables model accuracy and algorithmic validation, and AI validation, transparency, explainability, accountability and ethics.

In this regard, Catalonia has positioned AI as a strategic lever for health system transformation [24]. However, realizing its benefits depends not only on technical performance but also on whether models are reliable, representative and safe across diverse populations and care settings [74,75]. This is particularly relevant in a network-based publicly funded health care system such as Catalonia’s, where data are generated across heterogeneous providers, increasing the risk that AI models trained on historically shaped datasets reproduce existing patterns of access, diagnosis and treatment [76,77].

To mitigate these risks, Catalonia has developed system-level governance frameworks that extend AI validation beyond technical accuracy to include ethics, equity, transparency and continuous monitoring across the AI lifecycle [78-80]. These frameworks signal a shift toward conceiving algorithmic validation as an ongoing process linked to data quality, real-world performance and accountability rather than a one-off technical assessment. However, their existence does not guarantee equitable AI; evidence from AI-based digital health tools that have already been implemented at scale indicates that persistent challenges remain when translating validated solutions into routine practice. Even beyond pilot phases, limitations related to workflow integration, organizational readiness, and professional engagement continue to constrain effective use [81-84]. This illustrates that even in data-rich environments, effective AI governance must extend beyond technical validation to address organizational conditions for use, while publicly available evidence on fairness and equity-related performance across population subgroups remains limited.

Additional governance challenges arise from the incomplete institutional integration of AI-based tools into clinical information systems and regulated workflows. In the absence of officially embedded solutions, health professionals increasingly rely on general-purpose AI tools developed outside health care organizations [85,86], raising concerns regarding data protection, confidentiality, and accountability. From a governance perspective, this underscores how gaps in trusted, institutionally approved AI solutions may unintentionally displace responsibility onto individual practitioners.

Beyond implementation and integration challenges, equity-oriented AI validation in Catalonia is constrained by the limited availability of structured and routinely collected data capturing equity-relevant variables (eg, SDoH). Despite large volumes of clinical data, this gap limits both the assessment of algorithmic performance across population subgroups and the future integration of person-level DDoH into clinical information systems in a standardized and scalable manner [87].

In parallel, the recurrent pattern of pilot projects without sustained implementation suggests that the core challenge lies not only in developing or validating AI tools, but in embedding them within organizational and regulatory frameworks. In a diverse and rapidly expanding AI ecosystem such as Catalonia’s [22], these constraints underscore the need for system‑level coordination, shared validation standards, and robust governance mechanisms to support equitable and safe adoption.

### Axis 4

This axis includes personal customizability, inclusive design, and good practice design and focuses on how the design of digital health solutions shapes their effective, equitable, and sustained use.

In the Catalan context, these dimensions are particularly relevant given the growing role of patient-facing digital platforms [52,57-59]. While tools such as La Meva Salut represent an important step toward person-centered digital care, their largely standardized design and limited personalization options may constrain usability for users with lower digital or health literacy, disabilities, or reduced digital confidence [28]. Consistent with patterns discussed in earlier axes, differences in the uptake of digital health services across age groups and territories suggest that limited customizability may contribute to persistent inequalities in use [88-91].

While governance and certification frameworks ensure minimum standards of quality, safety and trust [24,26,92,93], guidance documents promoting inclusive and adaptable design principles have not yet been systematically translated into routine development and scaling practices [94]. The uneven translation of these principles suggests that inclusive and adaptive design remains encouraged but not yet structurally embedded across public digital health initiatives in Catalonia.

In addition, although inclusive design principles rely on meaningful user involvement, structured and sustained co-creation is not yet introduced as standard practice in Catalonia’s public digital health ecosystem. Participatory approaches remain largely project-based and tend to involve users with higher digital skills or closer links to innovation environments, limiting the representation of populations most exposed to digital exclusion [88,95-100].

Taken together, these observations reveal a gap between strategic commitments to inclusive, person-centered digital health and their operational implementation. Existing frameworks have strengthened quality and safety, but the limited structural integration of adaptive and co-creative design processes risks leaving inclusivity largely aspirational within Catalonia’s public digital health ecosystem.

### Axis 5

This axis includes security settings and features, privacy and security policy, data consent policy, access and sharing policy, and data governance and ethics, and examines how these variables shape the conditions under which digital health data are generated, shared, and reused within the health system.

In Catalonia, data governance is structured through a layered combination of European legal frameworks such as the General Data Protection Regulation, national and regional oversight, and technical safeguards, including certification and assessment frameworks [93,101-103]. In routine care, security mechanisms further support this data protection and are designed to protect personal data while supporting continuity of care across interconnected systems, but they also shape usability and clinical workflows, creating trade-offs between protection and access [104-107]. In fact, professional surveys indicate that limitations in formal interoperable systems may encourage reliance on informal communication channels, increasing risks for data protection and confidentiality [26].

From the citizen perspective, data governance challenges are compounded by limited transparency and control over how health data are accessed and used in routine practice. While data access is logged at the institutional level, patients generally lack user-friendly mechanisms to see who consults their data and for what purposes [108]. Recent European regulatory developments, notably the European Health Data Space (EHDS), explicitly acknowledge this gap by strengthening individual rights to access and oversight, although practical implementation at the regional level remains ongoing [109].

Beyond everyday data protection and access, the circulation of health data across organizational boundaries raises additional governance challenges related to consent, control, and secondary use. In Catalonia, strong data protection and ethical oversight frameworks safeguard public trust but also concentrate data use within specialized settings, potentially limiting its scalability and timeliness [64,110]. Complex procedures and perceived administrative and financial burdens can restrict access for actors outside data-holding institutions, reinforcing a model in which data control is primarily institutional rather than individual or system-wide [110]. Here too, the EHDS seeks to ease this tension by enabling secure secondary use without lowering protection standards [109].

Within this context, data consent emerges as a central and increasingly complex component of data governance. In highly digitalized systems, consent can no longer be understood as a one-time decision, increasing cognitive demands on individuals and raising concerns about consent fatigue and unequal capacity to exercise data rights over time [66,111]. Enhancing transparency and strengthening digital and data literacy may improve data autonomy and trust, while reducing the risk that existing governance arrangements disproportionately burden vulnerable groups [109].

Finally, operational threats such as cyberattacks highlight that data protection is not only a legal or procedural issue but also a matter of system resilience. Cybersecurity incidents further expose vulnerabilities in highly digitalized health systems, illustrating that effective data protection depends not only on regulatory compliance but also on organizational preparedness, coordination and workforce capacity [112-116].

### Axis 6

This axis focuses on the accountability and transparency of commercial interests in the interaction between publicly governed health systems and private actors involved in digital health.

While transparency is relatively well established at the individual-facing level, such as communication about service incidents, cybersecurity events, or platform changes [112-114], it is far less consistent in relation to decisions on the development, procurement, and integration of digital health technologies.

At the institutional level, public authorities have actively promoted collaboration with private actors through innovation programs, pilot projects, and public-private partnerships, particularly in areas such as digital platforms, data-driven tools and AI [70,117-119]. Although such collaborations are not inherently problematic, their integration into routine publicly funded care expands accountability beyond technical or clinical performance to include public value, power dynamics, long-term dependencies and responsibility for unintended effects [120].

In practice, these broader accountability demands are not matched by adequate transparency mechanisms. While contractual information is formally published [121], the absence of a searchable and standardized classification of digital health solutions limits system-wide oversight of what technologies are procured and under which conditions. Accountability therefore remains fragmented, reinforced by decentralized procurement and scaling processes across organizations and system levels. Existing certification and evaluation frameworks [78] provide important safeguards but are insufficient to address these wider governance challenges.

Addressing these gaps requires governance approaches that ensure transparency of commercial interests across the full lifecycle of digital health technologies, moving beyond postevaluation or contractual compliance.

### Implications for Governance and Health Planning

The findings of our study allow us to move beyond simply identifying which determinants, whether digital or determinants modified by digitalization, matter most for health in Catalonia, and to reflect on what they mean for how health systems should understand, govern and plan for digital transformation.

First, our analysis shows that, despite Catalonia’s advanced digital maturity and sustained public investment in digital health infrastructures [22-26], digitalization has not benefited all population groups equally [24,31,34]. This clearly reinforces the conclusions of previous studies indicating that DDoH have the capacity to generate, and not only to reduce, health inequalities [14-16,19,21,41]. A key question, therefore, would be which of the prioritized determinants are most likely to worsen these inequalities or disproportionately affect vulnerable populations. However, at present this question is difficult to answer with precision, since DDoH are not yet evaluated in a systematic way within health systems, and the available research tends to focus on person-related determinants, while more systemic determinants have received far less empirical scrutiny. As a result, the equity consequences of these system-level factors remain largely unexplored.

To this complexity is added the dual nature that these determinants seem to have. On the one hand, the prioritized determinants are clearly intersectional, interacting with one another and with broader social, commercial and political determinants. So, health inequalities may not simply accumulate but grow nonlinearly when multiple determinants intersect [14-16,41], which further complicates the identification of who is most affected and under which conditions. On the other hand, another important conclusion derived from our study is that health authorities may not be able to fully manage the determinants that matter most by themselves, since doing so is inherently intersectoral and will require coordinated action across domains that extend well beyond the health sector, including social services, education, telecommunications, digital administration and industry.

Beyond these governance implications, the results also offer an opportunity to return to a question raised at the outset of this paper: whether DDoH are genuinely new determinants of health, or whether they are simply the modern form in which SDoH now operate in increasingly digital societies. Indeed, several of the prioritized determinants have no clear equivalent before the digital age and cannot be fully explained by existing social categories, such as model accuracy, AI validation, or data governance. Others do not represent entirely new factors but rather transform how well-established social determinants, such as education, income, or place of residence, operate in a digital context. Hence, these findings support the view that DDoH are best understood as a domain in their own right, closely connected to the SDoH but not merely a digital version of them.

How, then, can these governance, equity and capacity considerations be embedded in health planning? In practical terms, DDoH could be systematically incorporated into several existing planning tools. First, they could be integrated into health needs assessments, so that digital factors are considered alongside social and clinical needs from the outset. Within strategic planning frameworks, this would ensure that priorities such as interoperability, data governance, or digital inclusion are explicitly addressed rather than assumed. Embedding them in monitoring systems would, in turn, allow health systems to track over time how digital factors affect access, quality, and equity. Finally, digital equity audits could help anticipate whether a given initiative is likely to narrow or widen existing inequalities before it is deployed. Incorporating DDoH into these instruments would provide a practical route for turning the priorities identified in this study into decisions and actions, and would help ensure that digital transformation contributes to reducing, rather than reinforcing, health inequalities.

### Limitations

A primary limitation of this study relates to the snowball sampling strategy that may have favored interconnected professional networks, and uneven representation across health regions limited territorial comparisons. However, these constraints are common in Delphi studies and, while they may introduce some selection bias, they are unlikely to invalidate the main consensus patterns identified.

Another limitation relates to the composition of the expert panel. Although we sought diversity, most participants were health system professionals, managers, researchers, and individuals with expertise in public health and digital health. The limited representation of actors from commercial sectors, broader political institutions, or civil society may have contributed to the lower emphasis placed on commercial and some political determinants. These patterns should therefore be interpreted as reflecting an expert‑system view of digital transformation within the Catalan public health system rather than a comprehensive representation of all stakeholder perspectives. Future exercises should incorporate patients, citizens and representatives from sectors beyond health in order to capture lived experience and intersectoral viewpoints more fully. Moreover, the study was not designed or powered to compare subgroups. We conducted exploratory analyses by sex, age, and professional role across all 127 determinants in Phase 1, but we did not find clear or consistent differences once we applied conservative corrections for multiple testing. Given the relatively small number of participants in each subgroup, our analyses cannot exclude the possibility that relevant differences would be detected in larger, specifically powered studies.

A further methodological limitation concerns the number of items included in the Delphi exercise. The assessment of 127 determinants may have contributed to respondent fatigue, potentially affecting attention and consistency. The approximately 28% attrition rate between the first and second rounds is likely to reflect, at least in part, the time demands and cognitive load associated with completing a lengthy survey. Furthermore, although all included participants completed ratings for all items, we cannot fully rule out fatigue‑related effects on rating quality. In principle, response duration data from Qualtrics could have been used to explore this issue, but participants were allowed up to three days to complete each round, so the recorded total duration often reflects extended elapsed time including breaks rather than actual answering time, and was therefore not a reliable indicator of completion time or fatigue.

At the same time, the exercise relied on definitions from the original framework [16]. Several definitions included illustrative examples drawn from the United States context, which were removed to improve relevance for Catalonia, and some determinants were framed primarily in relation to digital health interventions rather than their broader structural role. For example, socioeconomic inequalities may have been underrepresented as SDoH, a limitation acknowledged by experts during the final consensus meeting and reflected in Catalan policy priorities [24]. Nevertheless, we deliberately retained the full set of 127 determinants to reflect the breadth of the original framework and remain comparable with the global study. This choice involved accepting some conceptual and practical limitations, but avoided introducing additional selection bias.

Finally, the analysis of regional strategies, policy frameworks and other relevant documents was based on a purposive, narrative review rather than a systematic or scoping review, which may have led to the omission of some relevant documents. As a result, the mapping of existing initiatives may be incomplete, particularly for smaller-scale or locally implemented programs that were not identified, while the ongoing revision of key strategic documents and the rapid evolution of digital technologies further limit the temporal stability of the findings. The results should therefore be interpreted as a robust starting point rather than a definitive prioritization, to be refined through future studies with larger and more balanced samples.

### Conclusions

This study shows the value of systematically contextualizing DDoH and their impact to inform strategic health planning in digitally mature public health systems. By replicating and adapting a WHO-led global Delphi prioritization exercise to the Catalan context, we identified which digital and related health determinants are considered most important in this setting, examined how these priorities align with or differ from the global Delphi results, and connected them to existing regional strategies and policy documents. Taken together, our findings suggest that regional adaptation of global digital health priorities is needed to better reflect local infrastructures, governance arrangements and equity challenges.

Although Catalonia has advanced digital infrastructures and interoperable health information systems, experts identified key health determinants related to digital and health literacy, data governance, security and privacy, interoperability, inclusive design and the governance of AI-based tools. These findings indicate that, in digitally mature health systems, digital transformation should enter a new strategic phase in which governance and health equity considerations become central to planning, rather than secondary to technological expansion.

The proposed approach offers a replicable framework for translating international digital health agendas into regional planning processes, enabling health systems to move beyond technology-centered strategies toward more anticipatory, coordinated, and equity-oriented digital health governance. By systematically analyzing health determinants, this approach can support decision-makers in identifying structural risks, aligning digital transformation with population needs, and embedding equity considerations into strategic planning.

## Data Availability

The data generated during this study are provided in the supplementary materials. Multimedia Appendix 1 includes the sociodemographic questions and corresponding response categories used in the Delphi survey. Multimedia Appendix 2 presents the full set of assessed digital determinants of health together with their consensus outcomes.

## Appendix

Multimedia Appendix 1 Sociodemographic questions and response categories.

Multimedia Appendix 2 Full set of digital determinants of health and consensus outcomes.

## Abbreviations

AQuAS: Healthcare Quality and Assessment Agency of Catalonia
DDoH: digital determinants of health
EHDS: European Health Data Space
EHR: electronic health record
HES: Historial Electrònic de Salut
SDoH: social determinants of health
SIDIAP: Information System for the Development of Research in Primary Care
SISCAT: Integrated Public Healthcare System of Catalonia
WHO: World Health Organization

## References

[1] Nichols JA, Herbert Chan HW, Baker MAB (2019). Machine learning: applications of artificial intelligence to imaging and diagnosis. Biophys Rev.

[2] Elemento O (2020). The future of precision medicine: towards a more predictive personalized medicine. Emerg Top Life Sci.

[3] Alotaibi YK, Federico F (2017). The impact of health information technology on patient safety. Saudi Med J.

[4] Dave P (2024). How digital health is revolutionizing healthcare and contributing to positive health outcomes. J Drug Delivery Ther.

[5] Kitsiou S, Paré G, Jaana M, Gerber B (2017). Effectiveness of mHealth interventions for patients with diabetes: an overview of systematic reviews. PLoS One.

[6] Polisena J, Tran K, Cimon K, Hutton B, McGill S, Palmer K, Scott RE (2010). Home telemonitoring for congestive heart failure: a systematic review and meta-analysis. J Telemed Telecare.

[7] Eland-de Kok P, van Os-Medendorp H, Vergouwe-Meijer A, Bruijnzeel-Koomen C, Ros W (2011). A systematic review of the effects of e-health on chronically ill patients. J Clin Nurs.

[8] (2033). The world health assembly endorses the extension of the global strategy on digital health to 2027 and approves the next phase for 2028-2033. World Health Organization.

[9] (2023). Digital together: Germany's digitalisation strategy for health and care. Federal Ministry of Health (Bundesministerium für Gesundheit).

[10] (2021). Estrategia de salud digital del sistema nacional de salud [Report in Spanish]. Ministerio de Sanidad.

[11] (2024). France 2030 - Deux ans de la stratégie « santé numérique »: Faire de la France un leader en santé numérique [Report in French]. Gouvernement Français.

[12] (2024). Digital for care: a digital health framework for Ireland 2024-2030. Department of Health.

[13] (2022). Regional digital health action plan for the WHO European region 2023-2030 (EUR/RC72/5). World Health Organization, Regional Office for Europe.

[14] Kickbusch I, Piselli D, Agrawal A, Balicer R, Banner O, Adelhardt M, Capobianco E, Fabian C, Singh Gill A, Lupton D, Medhora RP, Ndili N, Ryś A, Sambuli N, Settle D, Swaminathan S, Morales JV, Wolpert M, Wyckoff AW, Xue L, Bytyqi A, Franz C, Gray W, Holly L, Neumann M, Panda L, Smith RD, Georges Stevens EA, Wong BLH (2021). The Lancet and Financial Times commission on governing health futures 2030: growing up in a digital world. The Lancet.

[15] Badr J, Motulsky A, Denis JL (2024). Digital health technologies and inequalities: a scoping review of potential impacts and policy recommendations. Health Policy.

[16] van Kessel R, Seghers LE, Anderson M, Schutte NM, Monti G, Haig M, Schmidt J, Wharton G, Roman-Urrestarazu A, Larrain B, Sapanel Y, Stüwe L, Bourbonneux A, Yoon J, Lee M, Paccoud I, Borga L, Ndili N, Sutherland E, Görgens M, Weicken E, Coder M, de Fatima Marin H, Val E, Profili MC, Kosinska M, Browne CE, Marcelo A, Agarwal S, Mrazek M, Eskanda H, Chestnov R, Smelyanskaya M, Källander K, Buttigieg S, Ramesh K, Holly L, Rys A, Azzopardi-Muscat N, de Barros J, Quintana Y, Spina A, Hyder A, Labrique A, Kamel Boulos M, Chen W, Agrawal A, Cho J, Klucken J, Prainsack B, Balicer R, Kickbusch I, Novillo-Ortiz D, Mossialo E (2025). A scoping review and expert consensus on digital determinants of health. Bull World Health Organ.

[17] Lyles CR, Nguyen OK, Khoong EC, Aguilera A, Sarkar U (2023). Multilevel determinants of digital health equity: a literature synthesis to advance the field. Annu Rev Public Health.

[18] Rice L, Sara R (2019). Updating the determinants of health model in the information age. Health Promot Int.

[19] Chidambaram S, Jain B, Jain U, Mwavu R, Baru R, Thomas B, Greaves F, Jayakumar S, Jain P, Rojo M, Battaglino MR, Meara JG, Sounderajah V, Celi LA, Darzi A (2024). An introduction to digital determinants of health. PLOS Digit Health.

[20] Jahnel T, Dassow HH, Gerhardus A, Schüz B (2022). The digital rainbow: digital determinants of health inequities. Digit Health.

[21] Duric P, Rajcevic S, Malbasa DJ, Mulic D (2026). What do we talk about when we talk about digital determinants of health? - A systematic review. Digit Health.

[22] (2025). Digital health in Catalonia. ACCIÓ Generalitat de Catalunya (Government of Catalonia).

[23] Piera-Jiménez J, Carot-Sans G, Ramiro-Pareta M, Nogueras MM, Folguera-Profitós J, Ródenas P, Jiménez-Rueda A, de Pando Navarro T, Mira Palacios JA, Fajardo JC, Ustrell Campillo J, Vela E, Monterde D, Valero-Bover D, Bonet T, Tarrasó-Urios G, Cantenys-Sabà R, Fabregat-Fabregat P, Gómez Oliveros B, Berdún J, Michelena X, Cano I, González-Colom R, Roca J, Solans O, Pontes C, Pérez-Sust P (2024). A 25-Year retrospective of health IT infrastructure building: the example of the Catalonia region. J Med Internet Res.

[24] (2021). Pla de salut de Catalunya 2021-2025 [Report in Catalan]. Generalitat de Catalunya, Departament de Salut.

[25] (2020). Monogràfic PADRIS 2020: Analítica de dades per a la recerca i la innovació en salut [Report in Catalan; archived version]. Agència de Qualitat i Avaluació Sanitàries de Catalunya (AQuAS).

[26] Piera-Jiménez J, Michelena VX, Climent FA, Valle L, Maymó-Costa A, Dordas A (2025). Visió col·lectiva per al futur de la salut: bases per a un nou model assistencial i de sistemes d?informació [Web page in Catalan]. Scientia.

[27] (2010). eHealth in Catalonia: we are connected [Report in Catalan]. Generalitat de Catalunya, Departament de Salut.

[28] (2023). La meva salut - fundació TIC salut i social [Web page in Catalan]. Generalitat de Catalunya, Fundació TIC Salut i Social.

[29] (2024). Història clínica compartida a Catalunya (HC3) [Web page in Catalan; archived version]. Generalitat de Catalunya, Departament de Salut.

[30] Gilabert-Perramon A, López-Calahorra P, Escoda-Geli N, Salvadó-Trias C (2010). Receta electrónica en Cataluña (Rec@t): una herramienta de salud [Article in Spanish]. Medicina Clínica.

[31] Fernández-Ardèvol M, Rodríguez E, Suárez-Gonzalo S (2025). Desconnectades? L’Índex Català de Bretxa digital: una eina per entendre i reduir les desigualtats digitals des de la societat civil i les administracions públiques [Report in Catalan]. Taula d’entitats del Tercer Sector Social de Catalunya.

[32] Fernández OS, Seguí FL, Vidal-Alaball J, Bonet Simo JM, Vian OH, Cabo PR, Hernandez MC, Dominguez CO, Reig XA, Rodríguez YD, Peralta MM, Hermosilla E, León NM, Guimferrer N, González MA, Cuyàs FG, Sust PP (2020). Primary care doctor characteristics that determine the use of teleconsultations in the Catalan public health system: retrospective descriptive cross-sectional study. JMIR Med Inform.

[33] Solans O, Vidal-Alaball J, Roig Cabo P, Mora N, Coma E, Bonet Simó JM, Hermosilla Pérez E, Saigí-Rubió F, Olmos Domínguez C, Piera-Jiménez J, Abizanda González M, López Seguí F (2021). Characteristics of citizens and their use of teleconsultations in primary care in the Catalan public health system before and during the COVID-19 pandemic: retrospective descriptive cross-sectional study. J Med Internet Res.

[34] Segur-Ferrer J, Moltó-Puigmartí C, Pastells-Peiró R, Sabadell E, Vivanco-Hidalgo RM (2023). Marco de evaluación de tecnologías sanitarias: adaptación para la evaluación de tecnologías de salud digital [Report in Spanish]. Ministerio de Sanidad, Agència de Qualitat i Avaluació Sanitàries de Catalunya (AQuAS).

[35] Davies AR, Honeyman M, Gann B (2021). Addressing the digital inverse care law in the time of COVID-19: potential for digital technology to exacerbate or mitigate health inequalities. J Med Internet Res.

[36] de Meyrick J (2003). The Delphi method and health research. Health Educ.

[37] World Medical Association (WMA) (2009). Declaration of Helsinki. Ethical principles for medical research involving human subjects. Jahrbuch für Wissenschaft und Ethik.

[38] Beiderbeck D, Frevel N, von der Gracht HA, Schmidt SL, Schweitzer VM (2021). Preparing, conducting, and analyzing Delphi surveys: cross-disciplinary practices, new directions, and advancements. MethodsX.

[39] Kirchherr J, Charles K (2018). Enhancing the sample diversity of snowball samples: recommendations from a research project on anti-dam movements in Southeast Asia. PLoS One.

[40] Škrinjar I, Vučić K, Puljak L (2026). Sample size and consensus methods used in Delphi studies are heterogeneous: meta-research study. J Clin Epidemiol.

[41] Kim KK, Backonja U (2025). Digital health equity frameworks and key concepts: a scoping review. J Am Med Inform Assoc.

[42] Barry J (2014). Don't be evil: should access to the internet be conceptualized as an instrumental human right?. SSRN Electron J.

[43] (2025). Central de Resultats. Dades del sistema de salut de Catalunya 2017-2024 [Report in Catalan]. Agència de Qualitat i Avaluació Sanitàries de Catalunya (AQuAS), Departament de Salut, Generalitat de Catalunya.

[44] Plaza A, Mas A (2025). L'orientació del nou mapa del sistema de salut de Catalunya: una aposta per la transformació i l'equitat. Annals de Medicina.

[45] Richardson S, Lawrence K, Schoenthaler AM, Mann D (2022). A framework for digital health equity. NPJ Digit Med.

[46] (2023). Pla sectorial per a la transició de Catalunya cap a l'Estat de benestar digital (2023-2027) [Report in Catalan]. Generalitat de Catalunya.

[47] (2024). Cercador de centres sanitaris [Web page in Catalan]. CatSalut. Servei Català de la Salut.

[48] (2025). Cartografia de la divisió territorial sanitària. Generalitat de Catalunya, Departament de Salut.

[49] Ruiz S (2025). Faltan 400 pediatras en la atención primaria de Cataluña [Web page in Spanish]. Cadena SER.

[50] (2023). Competencias digitales [Report in Spanish]. Observatorio Nacional de Tecnología y Sociedad (ONTSI), Red.es.

[51] Hernández Encuentra E, González Caballero JL, Montagni I, Fernández Gutiérrez M, Bas Sarmiento P (2025). Digital health literacy among the Spanish population: a descriptive and latent class analysis study. Eur J Public Health.

[52] eSalut - Germans trias. Generalitat de Catalunya.

[53] (2024). Més digitals [Web page in Catalan]. Polítiques Digitals.

[54] (2025). Programa pacient expert catalunya [Web page in Catalan]. Generalitat de Catalunya, Canal Salut.

[55] Bujan PA, González Mestre A, Blay Pueyo C, Ledesma Castelltort A, Contel Segura JC, Constante Beitia C (2015). Expert patient programme Catalonia: a person-centered community perspective. Int J Integr Care.

[56] Escola de salut Catalana [Web page in Catalan]. Generalitat de Catalunya.

[57] El Meu Vall d'Hebron.

[58] Portal Pacient SJD App. App Store.

[59] El meu HUB.

[60] Western MJ, Smit ES, Gültzow T, Neter E, Sniehotta FF, Malkowski OS, Wright C, Busse H, Peuters C, Rehackova L, Gabriel Oteșanu A, Ainsworth B, Jones CM, Kilb M, Rodrigues AM, Perski O, Wright A, König L (2025). Bridging the digital health divide: a narrative review of the causes, implications, and solutions for digital health inequalities. Health Psychol Behav Med.

[61] (2017). Pla Director de Sistemes d'Informació del SISCAT: construint junts una estratègia de salut digital per a Catalunya [Web page in Catalan]. Scientia.

[62] Solans O, Serra A, Hernandez S, Martinez J, Contel JC, Olmedo I, Sarquella E, Perez P, Garcia-Cuyas F (2018). Health and social electronic records integratation in Catalonia. Int J Integr Care.

[63] Interoperability and standards. Generalitat de Catalunya, Fundació TIC Salut i Social.

[64] Recalde M, Rodríguez C, Burn E, Far M, García D, Carrere-Molina J, Benítez M, Moleras A, Pistillo A, Bolíbar B, Aragón M, Duarte-Salles T (2022). Data resource profile: the information system for research in primary care (SIDIAP). Int J Epidemiol.

[65] Esteve-Matalí L, Vargas I, Amigo F, Plaja P, Cots F, Mayer EF, Pérez-Castejón JM, Vázquez ML (2021). Understanding how to improve the use of clinical coordination mechanisms between primary and secondary care doctors: clues from Catalonia. Int J Environ Res Public Health.

[66] Font AG (2014). 80 million documents now published in the shared medical record (HC3) in Catalonia. Fundació TIC Salut i Social.

[67] Piera-Jiménez J, Carot-Sans G (2025). Catalonia's journey to the open platform paradigm in healthcare. Digital Maturity in Hospitals.

[68] RETECH grants. Politiques Digitals.

[69] Catalonia.AI. Politiques Digitals.

[70] Programa PASS: Accede al sistema de salud de Cataluña [Web page in Spanish]. Biocat.

[71] Data centers in Barcelona are set to generate 7 billion euros by 2025. Catalonia Trade & Investment.

[72] López AB (2025). Los 'data centers' duplican su potencia en Barcelona y la consolidan como punto estratégico [Web page in Spanish]. The New Barcelona Post.

[73] Barcelona Supercomputing Center (BSC-CNS). Barcelona Supercomputing Center (BSC-CNS).

[74] Alderman JE, Palmer J, Laws E, McCradden MD, Ordish J, Ghassemi M, Pfohl SR, Rostamzadeh N, Cole-Lewis H, Glocker B, Calvert M, Pollard TJ, Gill J, Gath J, Adebajo A, Beng J, Leung CH, Kuku S, Farmer L, Matin RN, Mateen BA, McKay F, Heller K, Karthikesalingam A, Treanor D, Mackintosh M, Oakden-Rayner L, Pearson R, Manrai AK, Myles P, Kumuthini J, Kapacee Z, Sebire NJ, Nazer LH, Seah J, Akbari A, Berman L, Gichoya JW, Righetto L, Samuel D, Wasswa W, Charalambides M, Arora A, Pujari S, Summers C, Sapey E, Wilkinson S, Thakker V, Denniston A, Liu X (2025). Tackling algorithmic bias and promoting transparency in health datasets: the STANDING together consensus recommendations. The Lancet Digital Health.

[75] (2021). Ethics and governance of artificial intelligence for health: WHO guidance. World Health Organization.

[76] Model sanitari català [Web page in Catalan]. Generalitat de Catalunya.

[77] El SISCAT: sistema sanitari integral d'utilització pública de Catalunya [Web page in Catalan]. Catsalut.

[78] Moltó-Puigmartí C, Aussó-Trias S, Bretones VM, Domínguez HD, Vivanco-Hidalgo RM Assessment guide for digital health technologies that use artificial intelligence (AI). Generalitat de Catalunya.

[79] Health/AI programme. Generalitat de Catalunya, Fundació TIC Salut i Social.

[80] Eines d'IA en salut [Web page in Catalan]. Generalitat de Catalunya, Departament de Salut.

[81] Uns 1 Uns 1.200 lleidatans han utilitzat ja l'eina d'intel·ligència artificial per avaluar els seus símptomes abans d'anar a urgències [Web page in Catalan]. Institut Català de la Salut.

[82] Bellvitge posa a prova una eina pròpia d'IA per reduir cancel·lacions quirúrgiques i evitar proves innecessàries [Web page in Catalan]. Institut Català de la Salut.

[83] (2025). Salut es planteja estendre a tota Catalunya la transcripció amb IA de converses amb pacients [Web page in Catalan]. Diari de Barcelona.

[84] Temprana-Salvador J, López-García P, Castellví Vives J, de Haro L, Ballesta E, Rojas Abusleme M, Arrufat M, Marques F, Casas JR, Gallego C, Pons L, Mate JL, Fernández PL, López-Bonet E, Bosch R, Martínez S, Ramón Y Cajal S, Matias-Guiu X (2022). DigiPatICS: digital pathology transformation of the Catalan health institute network of 8 hospitals-planification, implementation, and preliminary results. Diagnostics (Basel).

[85] Neha F, Bhati D, Shukla DK, Amiruzzaman M (2024). ChatGPT: transforming healthcare with AI. AI (Basel).

[86] Blease C, Garcia Sanchez C, Locher C, McMillan B, Gaab J, Torous J (2025). Generative artificial intelligence in primary care: qualitative study of UK general practitioners' views. J Med Internet Res.

[87] Rodoreda-Pallàs B, Lumillo-Gutiérrez I, Miró Catalina Q, Torra Escarrer E, Sanahuja Juncadella J, Morin Fraile V (2023). Recording of social determinants in computerized medical records in primary care consultations: quasi-experimental study. JMIR Form Res.

[88] Veinot TC, Mitchell H, Ancker JS (2018). Good intentions are not enough: how informatics interventions can worsen inequality. J Am Med Inform Assoc.

[89] Czaja SJ, Charness N, Fisk AD, Hertzog C, Nair SN, Rogers WA, Sharit J (2006). Factors predicting the use of technology: findings from the center for research and education on aging and technology enhancement (CREATE). Psychol Aging.

[90] Zeeb H, Maaß L, Schultz T, Haug U, Pigeot I, Schüz B (2025). Digital Public Health: Interdisciplinary Perspectives.

[91] Hollimon LA, Taylor KV, Fiegenbaum R, Carrasco M, Garchitorena Gomez L, Chung D, Seixas AA (2025). Redefining and solving the digital divide and exclusion to improve healthcare: going beyond access to include availability, adequacy, acceptability, and affordability. Front Digit Health.

[92] Declaración de accesibilidad del Área privada ciudadana [Web page in Spanish]. Generalitat de Catalunya.

[93] Marco de evaluación de activos digitales [Web page in Spanish]. Generalitat de Catalunya, Fundació TIC Salut i Social.

[94] (2025). Good practice guide for developing digital assets for citizens. Generalitat de Catalunya, Fundació TIC Salut i Social.

[95] (2024). i2cat. ENoLL (European Network of Living Labs).

[96] Piera-Jiménez J, Vliarasau N, Maymó A, Michelena X, Climent FA, Farré A Co-creating principles for digital health equity: a participatory human-centred design approach for system-level transformation across Catalonia's health ecosystem. JMIR Preprints.

[97] Actividades de co-creación [Web page in Spanish]. Healthcare Living Lab Catalonia.

[98] Baltaxe E, Cano I, Risco R, Sebio R, Dana F, Laxe S, Martínez R, Ozores F, Roca J, Martínez-Pallí G (2022). Role of co-creation for large-scale sustainable adoption of digitally supported integrated care: prehabilitation as use case. Int J Integr Care.

[99] Dearden A, Haider RS (2015). ICT4D and participatory design. The International Encyclopedia of Digital Communication and Society.

[100] Schou J, Pors AS (2018). Digital by default? A qualitative study of exclusion in digitalised welfare. Soc Policy Adm.

[101] Agencia Española de Protección de Datos (AEPD) [Web page in Spanish]. Agencia Española de Protección de Datos.

[102] Autoritat Catalana de Protecció de Dades [Web page in Catalan]. Autoritat Catalana de Protecció de Dades (APDCAT).

[103] Data protection officer. European Data Protection Board (EDPB).

[104] (2020). Tecnologías y protección de datos en las AA.PP. [Report in Spanish]. Agencia Española de Protección de Datos.

[105] Privacidad y seguridad [Web page in Catalan]. CPS - La Meva salut.

[106] Cybersecurity of hospitals and healthcare providers. European Commission.

[107] Governança, innovació i seguretat TIC en salut [Web page in Catalan]. Generalitat de Catalunya.

[108] The new EU Regulation on the protection of personal data: what does it mean for patients?. European Patients' Forum.

[109] European health data space regulation (EHDS). European Comission.

[110] SIDIAP data base. IDIAP Jordi Gol.

[111] (2019). Health in the 21st century: putting data to work for stronger health systems. Organisation for Economic Co-operation and Development.

[112] Sala J (2025). Ciberataque con ransomware al sistema Logaritme en Cataluña [Web page in Spanish]. Infordisa/Security Operations Center.

[113] Punzano D (2025). La sanidad se 'acostumbra' al chantaje de los hackers: 'No hay que pagar' [Web page in Spanish]. Redacción Médica.

[114] Ricart M (2022). Un ciberataque de los que piden rescate afecta al consorci sanitari integral [Web page in Spanish]. La Vanguardia.

[115] Coll B (2024). La Generalitat concluye que el Clínic no tenía medidas de seguridad 'mínimas' para contener el ciberataque de 2023 [Web page in Spanish]. Ediciones EL PAÍS SL.

[116] (2023). Ciberataque ransomware paraliza actividad del hospital clínic de Barcelona [Web page in Spanish]. Incibe-Cert.

[117] (2021). Inteligencia Artificial (IA): promoción y desarrollo de la IA en el Sistema de Salud de Catalunya [Web page in Spanish]. Generalitat de Catalunya, Departament de Salut.

[118] Health tech forward 2025 strengthens Catalonia's leadership in digital health. Biocat.

[119] Proyectos en colaboración público-privada 2025 [Web page in Spanish]. Agencia Estatal de Investigación.

[120] Denis JL (2014). Accountability in healthcare organizations and systems. Healthc Policy.

[121] (2024). Catàleg de portals de la transparència de Catalunya [Web page in Catalan]. Dades Obertes Catalunya.

