# Supplementary material for: Assessing Digital Determinants of Health and Their Impact as Part of Strategic Health Planning in Catalonia: Modified Delphi Study
Source: J Med Internet Res. 2026 Oct 29;28:e93840. doi: 10.2196/93840 (PMC13760322; doi:10.2196/93840)
Supplement: Supplementary file 1 [file jmir_v28i1e93840_app1.docx]

# Appendix A. Sociodemographic questions and response categories

| **VARIABLE** | **QUESTION** | **RESPONSE TYPE AND CATEGORIES** |
| --- | --- | --- |
| Age | What is your age? | Open text (numerical answer) |
| Gender | What is your gender? | Multiple choice with single answer:   - Male - Female - Others - Prefer not to say |
| Educational level | What is your maximum educational level achieved? | Multiple choice with single answer:   - Mid-level vocational training - Higher-level vocational training - Bachelor degree - Master's degree - PhD degree |
| Professional Background / Title | Which mid-level, higher-level, or university degree do you hold? | Open text |
| Current professional role | Indicate your current professional role | Open text, but afterwards grouped into three broad categories (Clinical role, Research/Academia, and Leadership/Management) based on the primary function described by participants. |
| Sector | What is your employment sector? | Multiple choice with single answer:   - Public healthcare sector - Private healthcare sector - Mixed public–private healthcare sector - Research - University - Other (with open text specification) |
| Years of experience in healthcare | How many years have you been working in the health sector? | Open text (numerical answer) |
| Years of experience in public health | How many years have you been working in the public health sector? | Open text (numerical answer) |
| Years of experience in digital health | How many years have you been working in the digital health sector? | Open text (numerical answer) |
| Work health region | In which health region do you primarily carry out your work activity? | Multiple choice with single answer:   - Alt Pirineu i Aran Health Region - Lleida Health Region - Camp de Tarragona Health Region - Terres de l'Ebre Health Region - Central Catalonia Health Region - Girona Health Region - Barcelona City Health Region - Barcelona North Metropolitan Health Region - Barcelona South Metropolitan Health Region - Penedès Health Region - Catalonia |
| Self-rated expertise on SDoH | What level of knowledge do you think you have about the SDoH? | 11-point Likert scale (0: No knowledge – 10: Extensive knowledge) |
| Self-rated expertise on DDoH | What level of knowledge do you think you have about the DDoH? | 11-point Likert scale (0: No knowledge – 10: Extensive knowledge) |
